# DIFFERENTIAL EFFECTS OF STATINS ON COGNITION IN WOMEN AT RISK FOR ALZHEIMER’S DISEASE

**DOI:** 10.1093/geroni/igz038.3171

**Published:** 2019-11-08

**Authors:** Tonita E Wroolie, Siena Roat-Shumway, Katie Watson, Natalie Rasgon

**Affiliations:** 1 Stanford University, School of Medicine, Department of Psychiatry & Behavioral Sciences, Stanford, California, United States; 2 Stanford University, School of Medicine, Stanford, California, United States

## Abstract

It is well established that the apolipoprotein epsilon 4 allele (APOE4) and being female are risk factors for late onset Alzheimer’s disease (AD) and declines in verbal learning and memory are early cognitive symptoms of conversion to AD. Because of conflicting findings regarding the effects of statins on cognition, this study examined statin use with respect to verbal learning and memory by APOE4 status in a sample of cognitively unimpaired women at risk for AD. Neuropsychological, statin use, and APOE4 data were utilized as a secondary analysis from an ongoing longitudinal study at the Banner Alzheimer’s Institute in Arizona. Subjects were cognitively unimpaired women aged 47-75 with a family history of probable AD in at least one first-degree relative. Neuropsychological outcome variables included total learning, immediate memory, and delayed memory scores from the Rey Auditory Verbal Learning Test (RAVLT). Statin use was defined by use of a cholesterol lowering drug at study enrollment. APOE4 status was defined by presence of at least one APOE4 allele. Linear regression analyses were conducted to determine existence of interactions between statin use and APOE4 status on cognition. Statistically significant interactions were found between statin use and APOE4 status in RAVLT total learning and immediate memory. Statin use in women APOE4 non-carriers was associated with better verbal learning and immediate memory performances whereas statin use in women APOE4 carriers was associated with worse performances on these same tasks. Conclusions. Findings suggest that sex and APOE4 status may be important factors in consideration of statin use.

